# Personalized Oncogenomics: Clinical Experience with Malignant Peritoneal Mesothelioma Using Whole Genome Sequencing

**DOI:** 10.1371/journal.pone.0119689

**Published:** 2015-03-23

**Authors:** Brandon S. Sheffield, Anna V. Tinker, Yaoqing Shen, Harry Hwang, Hector H. Li-Chang, Erin Pleasance, Carolyn Ch’ng, Amy Lum, Julie Lorette, Yarrow J. McConnell, Sophie Sun, Steven J. M. Jones, Allen M. Gown, David G. Huntsman, David F. Schaeffer, Andrew Churg, Stephen Yip, Janessa Laskin, Marco A. Marra

**Affiliations:** 1 University of British Columbia, Department of Pathology and Laboratory Medicine, Vancouver, Canada; 2 British Columbia Cancer Agency, Division of Medical Oncology, Vancouver Centre, Vancouver, Canada; 3 Canada’s Michael Smith Genome Sciences Centre, British Columbia Cancer Agency, Vancouver, Canada; 4 PhenoPath Laboratories, Seattle, Washington, United States of America; 5 University of British Columbia, Department of Surgery, Surgical Oncology, Vancouver, Canada; Virginia Commonwealth University, UNITED STATES

## Abstract

Peritoneal mesothelioma is a rare and sometimes lethal malignancy that presents a clinical challenge for both diagnosis and management. Recent studies have led to a better understanding of the molecular biology of peritoneal mesothelioma. Translation of the emerging data into better treatments and outcome is needed. From two patients with peritoneal mesothelioma, we derived whole genome sequences, RNA expression profiles, and targeted deep sequencing data. Molecular data were made available for translation into a clinical treatment plan. Treatment responses and outcomes were later examined in the context of molecular findings. Molecular studies presented here provide the first reported whole genome sequences of peritoneal mesothelioma. Mutations in known mesothelioma-related genes *NF2*, *CDKN2A*, *LATS2*, amongst others, were identified. Activation of *MET*-related signaling pathways was demonstrated in both cases. A hypermutated phenotype was observed in one case (434 vs. 18 single nucleotide variants) and was associated with a favourable outcome despite sarcomatoid histology and multifocal disease. This study represents the first report of whole genome analyses of peritoneal mesothelioma, a key step in the understanding and treatment of this disease.

## Introduction

### Peritoneal Mesothelioma

Mesotheliomas are malignancies arising from the serous cells forming the mesothelial linings of the pleura, pericardium, and peritoneum. The pleura is the most common site of diagnosis and accounts for the majority of mesotheliomas, while approximately 30% of cases are primary peritoneal in origin.[1] Peritoneal mesotheliomas in the general population are rare, with an annual incidence of 200–400 cases per year in the United States.[2] Risk factors for mesothelioma include occupational or industrial exposure to amphibole type asbestos fibers, genetic predisposition, and radiation.[3] The risk factors for pleural and peritoneal mesotheliomas are similar; however, asbestos exposure is more frequently associated with pleural disease.

There are three histologic subtypes of mesothelioma: epithelioid, sarcomatoid, and biphasic (mixed epithelioid/sarcomatoid). Epithelioid forms of primary peritoneal mesothelioma treated with cytoreductive surgery and hyperthermic intraperitoneal chemotherapy have reasonably good prognosis, with 5 year survival rate (possibly cures) of 70% for women and 40% for men.[4] Purely sarcomatous mesotheliomas are rare and sarcomatoid differentiation typically portends a poor prognosis.[5]

Molecular understanding of this rare entity has lagged behind the more prevalent human cancers. Much of the molecular information regarding peritoneal mesotheliomas is extrapolated from their pleural counterparts. Homozygous deletion of the 9p21 region has been described in a subset of body cavity mesotheliomas and can be useful for diagnosis and prognosis.[6] This deletion results in the simultaneous loss of *CDKN2A* (p16) and *ARF* (p14), two key regulators of the cell cycle. Similarly, the *BRCA-associated protein 1 (BAP1)* gene at 3p21 is also commonly lost or inactivated in mesothelioma.[7] Germline mutations in *BAP1* are known to predispose carriers to mesothelioma,[8] and other malignancies such as uveal melanoma and clear cell renal carcinoma.[9]

Loss of the tumor suppressors *NF2* and *LATS2* have both been described in pleural mesotheliomas[10]. *LATS2* transcribes a regulatory protein in the Hippo signaling pathway, involved in the determination of organ size through the regulation of cellular proliferation and apoptosis. Over-expression of anti-apoptotic factors such as *BCL-XL* has been postulated to play a role in mesothelioma pathogenesis.[11] Several receptor tyrosine kinases, including *EGFR*, *PDGFR*, *KIT*, *VEGFR*, and *MET* may drive mesothelioma growth; however attempts at targeted therapy towards these receptors have been unsuccessful.[12] Whole-genome analyses of pleural mesothelioma have identified a number of single-nucleotide variations (SNVs), as well as gross chromosomal alterations.[13] To our knowledge, results from whole-genome analysis of peritoneal mesothelioma have not been previously reported.

### Personalized OncoGenomics

The Personalized OncoGenomics project at the British Columbia Cancer Agency (Vancouver, Canada) is an ongoing study evaluating the use of comprehensive molecular analyses to guide diagnosis and treatment of patients with advanced malignancies. Patients are referred to the program by their treating oncologists, and are selected based upon tumor type, performance status, and limited treatment options.

Molecular workup is performed on one or more fresh-frozen tumor biopsies obtained specifically for molecular analyses, as well as archival tissue, typically FFPE (formalin-fixed, paraffin-embedded) blocks and a peripheral blood sample as a germline DNA reference. Molecular analyses include targeted deep sequencing of a panel of cancer-related genes, whole-genome sequencing, and gene expression profiling using RNA Seq (performed on fresh-frozen biopsies only). Data are processed using a well-established bioinformatics pipeline. Each case is presented at a multi-disciplinary conference with oncologists, molecular pathologists, bioinformaticians, and basic scientists. Clinical history, imaging, pathology, and molecular results are reviewed and clinically actionable findings are discussed. The aim of this approach is to provide patients and their oncologists with rational treatment options by linking genomic data with available targeted therapies or active clinical trials.

To date, more than 100 patients have participated in the Personalized OncoGenomics (POG) study. POG analyses have provided accurate and definitive diagnosis in instances where conventional clinical, radiological, and pathological methods have failed.[14] From a research perspective, the program has led to the first whole genome sequences of rare tumor types such as appendiceal adenocarcinoma, and in this report, peritoneal mesothelioma. In this report, we review the clinical history and molecular findings from two patients with primary peritoneal mesothelioma and discuss how the POG approach may offer new insights into the biology and clinical management of this difficult to treat disease.

## Results

### Case 1

**Fig 1:**
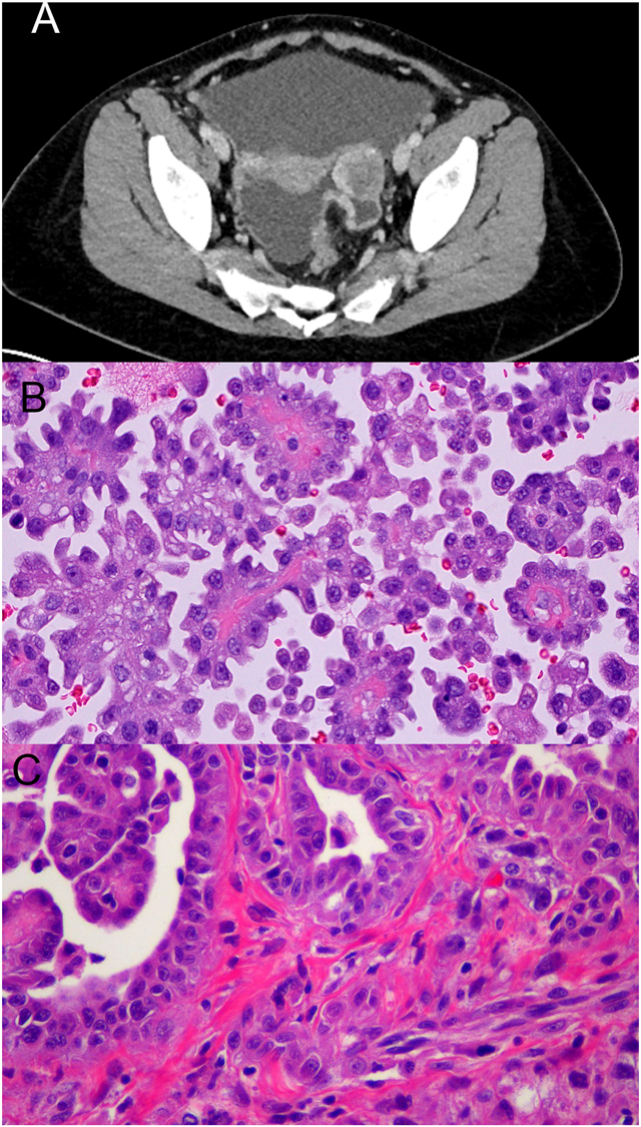
Patient 1 pertinent radiology and pathology. (A)Axial CT scan showing peritoneal-based pelvic mass. (B) Abdominal fluid aspiration biopsy showing papillary mesothelial proliferation (H&E stain on cell block preparation, 40X magnification). (C) Omental disease sampled at cytoreductive surgery showing malignant mesothelial cells forming gland-like structures, papillae, with spindle cell formation and sarcomatoid features. No treatment effect is appreciable (H&E 40X).

Patient 1, a young woman, presented to medical attention with a 3-month history of bloating, nausea, change in bowel habits, and night-sweats. There was no reported history of asbestos exposure, and no contributory family history. Cross-sectional imaging identified a peritoneal mass (Fig. 1a). Core needle biopsy and peritoneal cytology confirmed the diagnosis of epithelioid mesothelioma (Fig. 1b). The patient underwent 3 cycles of systemic chemotherapy with cisplatin and pemetrexed with modest response. Shortly after the initiation of chemotherapy, a fresh-frozen biopsy from a tumor deposit in the omentum was obtained for molecular analysis. The patient then underwent cytoreductive surgery and hyperthermic intraperitoneal chemotherapy. Histology from the cytoreductive surgery specimen showed a biphasic mesothelioma invading multiple abdominal and pelvic viscera with high-grade cytology and no appreciable treatment effect (Fig. 1c).

The FFPE material from the original diagnostic biopsy was exhausted during workup and thus molecular analysis was performed on the fresh-frozen omental biopsy (high cellularity, 59% tumor content), as well as peripheral blood. Targeted panel sequencing did not identify any significant mutations. Whole-genome sequencing (107X tumor and 40X germline depth of coverage) identified a somatic frameshifting mutation in *NF2* (Y177fs, COSM23800), as well as somatic SNVs in *SOCS3* (N92K), an inhibitor of *JAK2* tyrosine kinase;[15] *TET3* (R870G), a gene involved in chromatin remodeling;[16] and *TFPT* (G128W) a promoter of apoptosis (Table 1).[17] One insertion/deletion, 13 additional SNVs, and 16 gross chromosomal aberrations were identified. Assessment of the *BAP1* locus revealed wild-type alleles in both the tumor and germline.

**Table 1:** Select SNVs from patients 1 and 2.

Patient 1	Patient 2
***CNTNAP3B* M1247I**	*CDKN2A* R58*
***NF2* Y177fs**	***CNTNAP3B* M1247I**
*SOCS3* N92K	*LATS2* R958H
*TET3* R870G	*NF2* R466*
*TFPT* G128W	*NOTCH1* P2097L
	*SMO* V210M
	*TP53* R213*

*NF2* loss-of-function mutations were common to both patients, as well as mutations in *CNTNAP3B* (shown in bold). For a complete list of SNVs see S1 File and S2 File.

RNA expression analysis detected increased expression of *MET* and *NOTCH1* compared to both normal controls and tumor expression data from a range of malignancies publicly available from The Cancer Genome Atlas (TCGA). A fluorescent *in situ* hybridization (FISH) assay directed at the *MET* locus did not demonstrate amplification. FISH for possible 9p21 deletion showed intact loci. Immunohistochemical (IHC) analysis of BAP1 protein showed intact nuclear expression in tumor cells. FISH and IHC assays were performed on FFPE sections from the omental tumor sample obtained at surgery. Pathway analysis conducted based upon a synthesis of available data revealed deregulation of the NOTCH and PI3K-mTOR pathways (S1 Fig).

Within two months of cytoreductive surgery, the patient progressed clinically and radiographically. She was retreated with platinum doublet chemotherapy with no response. Based upon molecular results, the patient was briefly treated with everolimus with no response, deterioration continued and the patient died from progressive disease. For a complete set of genomic alterations and RNA expression data, see S1 File.

### Case 2

**Fig 2:**
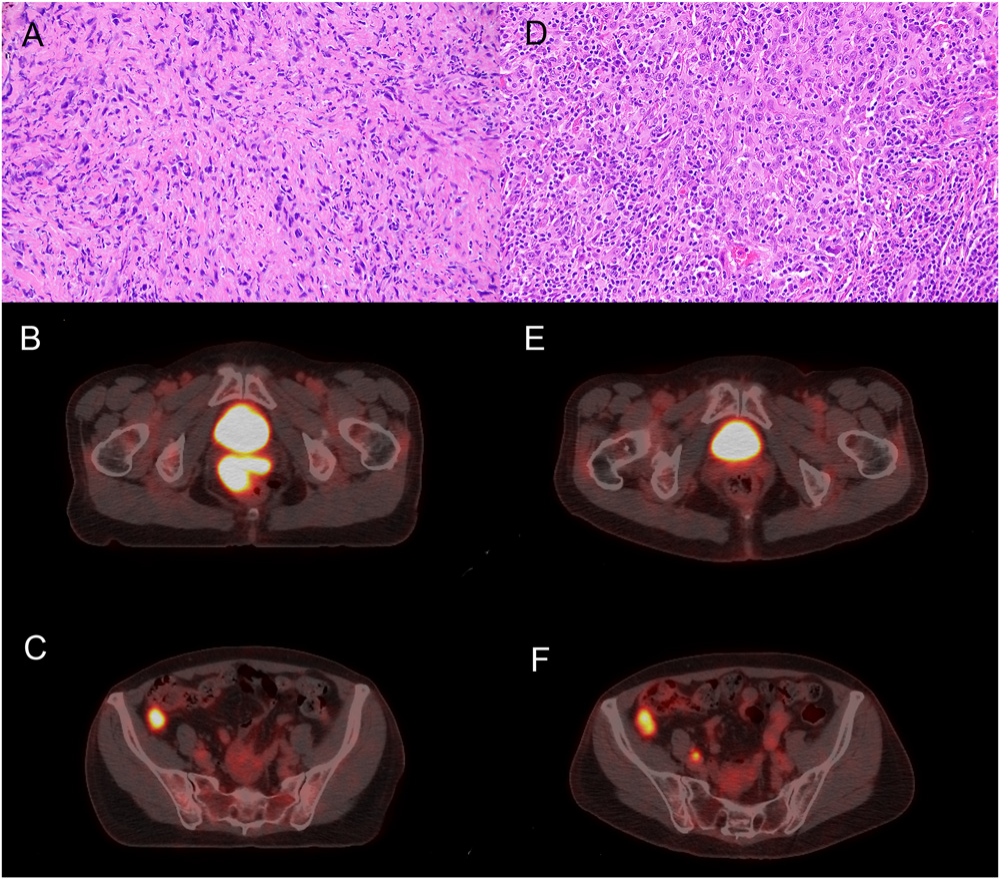
Patient 2 pertinent radiology and pathology. (A)Vaginal mass biopsy showing malignant mesothelial cells with sarcomatoid features (H&E 20X). (B) CT/PET scan showing uptake in vaginal mass. (C) CT/PET showing additional uptake in the cecum. (D) Surgical specimen from the cecum showing epithelioid mesothelial proliferation with heavy lymphoplasmacytic infiltrate (H&E 20X). (E) Post-treatment CT/PET showing resolution of the vaginal mass (as compared to B). (F) Post-treatment CT/PET showing persistence of the cecal mass (compared with C). Imaging of associated inguinal lymphadenopathy not shown.

Patient 2 was a middle-aged woman with a prior hysterectomy, no history of asbestos exposure and no significant family history. She presented with a vaginal wall mass, biopsy of which showed a sarcomatoid mesothelioma (Fig. 2a). Imaging by PET CT revealed several additional peritoneal lesions, including one adjacent to the right colon (Fig. 2b,c). Systemic chemotherapy was initiated with carboplatin and taxol. After 4 cycles, a discordant response was observed with complete resolution of the vaginal mass (Fig. 2e) but persistence of the right colonic nodule (Fig. 2f). CT-guided biopsy of this residual nodule showed a sarcomatoid mesothelioma, in keeping with the histology of the vaginal mass. After 6 cycles of carboplatin and taxol, the patient underwent cytoreductive surgery with hyperthermic intraperitoneal chemotherapy, during which all sites of persistent and presumed regressed disease were resected. A fresh-frozen biopsy of the right colonic nodule was obtained intraoperatively for molecular workup. Final pathology from the surgical specimens showed epithelioid mesothelioma in the right colonic nodule—no sarcomatoid component was identified (Fig. 2d). Scar tissue, indicative of treated disease, replaced the previously biopsied vaginal mass.

Molecular analyses were performed on FFPE tissue from the original diagnostic biopsy of the vaginal mass (high cellularity, 70% tumor content), as well as the fresh-frozen intraoperative biopsy of the right colonic nodule (high cellularity, 56% tumor content). Targeted panel sequencing identified a missense mutation in the *smoothened* gene (*SMO* V210M, COSM364081), a member of the hedgehog signaling pathway previously implicated in pleural mesothelioma.[18] A nonsense mutation in *p53* (R213*, COSM10654), and a nonsense mutation in *CDKN2A* (R58*, COSM12473); the mutation in *CDKN2A* was identified only in the chemotherapy-resistant right colon nodule. IHC directed towards p16 displayed no immunoreactivity in either tumor sample. BAP1 IHC demonstrated nuclear expression in tumor cells. FISH did not demonstrate loss of chromosome 9p21.

**Fig 3:**
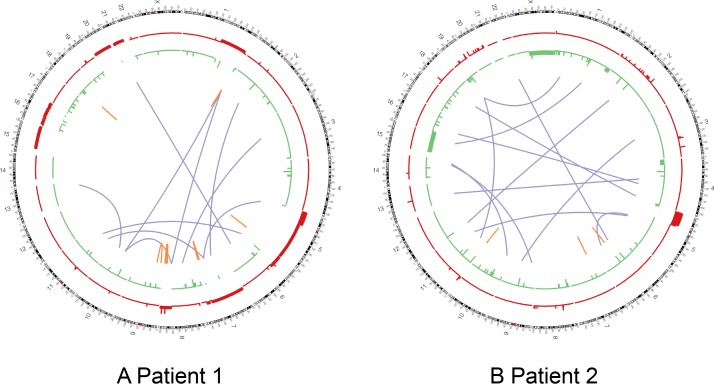
Patient 1 and 2 large-scale genomic alterations. Circos Plots for patients 1 (A) and 2 (B). Copy number gains shown in red, copy number losses shown in green (height of bars is proportional to the number of copies gained or lost). Purple lines indicate interchromosomal translocations. Orange lines indicate intrachromosomal translocation.

Whole genome sequencing (106X frozen intraoperative biopsy, 41X FFPE diagnostic biopsy, 48X germline depths of coverage) of both specimens confirmed all of the variants identified by panel sequencing. Additionally, a nonsense mutation in *NF2* (R466*, COSM23667) was identified in both samples. A novel missense mutation in *LATS2* (R958H) was identified in both samples, affecting a highly conserved residue of the tumor suppressor protein. Another novel missense mutation in *NOTCH1* (P2097L) was also identified in both samples affecting a highly conserved region of the cell-surface receptor that has also been previously implicated in mesothelioma by activating the PI3K-mTOR cell-signaling pathway (Table 1).[19] In total, 116 somatic coding SNVs were identified in both tumor samples, 151 additional coding SNVs were uniquely identified in the vaginal tumor, and 167 coding SNVs were uniquely identified in the pericolic tumor indicating a clear clonal divergence of the two malignant cell populations, further demonstrated by their differing histotypes. Nine large-scale structural variants were identified in the pericolic tumor on whole-genome sequencing (Fig. 3).

RNA expression data, together with genome sequence data, were used to perform a pathway analysis that showed deregulation of the Hippo and PI3K-AKT pathways. Upregulation of the *MET* tyrosine kinase was observed, and confirmed with 3+ immunohistochemical reactivity, but FISH did not demonstrate amplification at the *MET* locus (S2 Fig).

In the setting of a hypermutated tumor, we analyzed the molecular data to identify a candidate mutational signature.[20] All coding and noncoding SNVs as well as adjacent base pairs were analyzed showing a predominance of cytosine to thymine (C>T) transitions, a signature associated with cytosine deamination, thought to be associated with aging.[21]

The patient remains clinically well and disease free for 14 months following surgery. For a complete set of genomic alterations and RNA expression data, see S2 File.

## Discussion

Peritoneal mesothelioma is a rare malignancy that is difficult to diagnose and treat. Recent advances have led to improved outcomes through a combination of systemic chemotherapy, cytoreductive surgery and hyperthermic intraperitoneal chemotherapy. An understanding of the molecular biology of this disease is growing, but has not yet led to effective targeted therapy. The Personalized OncoGenomics initiative at the British Columbia Cancer Agency provides a unique opportunity to learn how to best analyze, interpret and apply rapidly emerging molecular information in the clinic. This approach to personalized medicine is in its infancy, and there is much to be learned from the wealth of molecular data generated, particularly in rare tumor types.

Comparing patients 1 and 2, similarities include loss-of-function mutations in *NF2*, a well-characterized tumor suppressor gene that is frequently inactivated in mesotheliomas. Interestingly, high expression of the *MET*, tyrosine kinase receptor was observed in both cases, with subsequent dysregulation of the downstream PI3K-mTOR pathway. The possibility of targeted treatment towards this cell-signaling pathway is an attractive option, and has been discussed and explored by others.[22,23,24] A single common mutation in the *contactin associated protein-like 3B* (*CNTNAP3B*, M1247I, COSM1472297) gene was shared between the two patients; the significance of this is not clear. Mutations in this gene have been reported to occur at low frequencies in tumors of the thyroid (0.98% of cases tested), prostate (0.38% of cases), and lung (0.21% of cases).[25]

The clinical and molecular differences between the two cases were notable. Patient 1 presented at a younger age, with significant clinical symptoms, a high disease burden and epithelioid histology, yet responded poorly to therapy and died within 8 months of diagnosis. In contrast, patient 2 presented with minimal clinical symptoms, was diagnosed with a poor prognosis sarcomatoid peritoneal mesothelioma, but had an excellent response to treatment, and remains clinically well and disease-free following multimodality therapy. This somewhat paradoxical excellent response to platinum-based chemotherapy in a patient predicted to have poor prognosis has been previously reported in the literature, also in a patient with sarcomatoid peritoneal mesothelioma.[26] Based upon this report and one other, sarcomatoid histology on biopsy may not necessarily exclude the possibility of a favorable outcome, suggesting that peritoneal mesothelioma of sarcomatoid histology should not automatically exclude patients from aggressive medical and surgical treatment. Additional studies are warranted to further explore this possibility.

The molecular data from patient 2 were probed extensively for possible explanations for the brisk response to therapy. The chemotherapy-responsive component of the tumor showed sarcomatoid histology and contained 151 unique mutations, while the chemotherapy-resistant component showed an epithelioid histology with 167 unique mutations. Subsets of genetic changes present in the responsive and nonresponsive elements were examined for any possible mechanism for chemotherapy resistance or sensitivity without success.

It is notable that when comparing whole genome sequencing data from the two patients, only 18 SNVs were identified in the tumor samples from patient 1, as compared to 434 SNVs in the samples from patient 2. This is a striking difference, even when accounting for the fact that patient 2 had an additional tumor sample submitted for molecular analysis. These findings suggest that a hypermutated phenotype of peritoneal mesothelioma may exist, similar to previously described mismatch-repair unstable colorectal cancers, or tobacco-associated lung cancers.[27]

Hypermutation may be defined as a subset of tumors that show an increased rate of mutation as compared to their non-hypermutated counterparts. In peritoneal mesothelioma, there is a paucity of information regarding the overall mutation rate of these tumors, making a strict definition difficult. In colorectal carcinoma, a well-studied example of hypermutation, mutation rates greater than 12/megabase (Mb) have been previously used as cutoffs.[28] By this cutoff, neither of these cases would be considered hypermutated (mutation rates of 0.8/Mb and 5.7/Mb in patients 1 and 2 respectively). Alternatively, in a recent review of hypermutation, Roberts and Gordenin considered a cutoff of greater than 10,000 single nucleotide changes,[29] a cutoff that includes patient 2 (16,329 single nucleotide changes identified). Many factors come into play when establishing a strict definition for hypermutation, for example the proliferation rate of the tumor, and the method of DNA sequencing.

In patient 2, somatic hypermutation was associated with platinum sensitivity and favorable prognosis, similar to findings from studies in other tumor types.[30] Other frequently hypermutated cancers are associated with defects in DNA repair machinery, or ongoing mutagenesis by an external carcinogen. Neither a DNA-repair defect nor a source of ongoing inflammation or carcinogen exposure was identified in the genomic data or clinical history of patient 2. The right-colonic tumor sample from patient 2 was obtained after 6 cycles of platinum-based chemotherapy. Although possible that the observed number of mutation is elevated secondary to chemotherapeutic effect, this is unlikely as the number of mutations in the pretreated vaginal sample is similar (283 and 267 coding mutations respectively). Analysis of the 283 coding SNVs, as well as 16,046 noncoding SNVs from the right colonic tumor sample, and their flanking base pairs was performed and compared to the distinct mutational signatures presented in Alexandrov *et al*.[21]. This analysis showed a prevalence of C>T transitions at NCG trinucleotide sequences, a mutational signature related to cytosine deamination which is observed in a wide variety of tumor types and not known to be associated with a specific etiology. No known chemotherapy-induced or DNA-repair deficiency hypermutation signatures were identified.

In conclusion, the elucidation and publication of whole-genome sequencing data represent a crucial milestone in the understanding of peritoneal mesothelioma. Data presented in this report provide further evidence for the role of tumor suppressor genes such as *CDKN2A*, *NF2* in the pathogenesis of peritoneal mesothelioma, and presents several other mutations that may also play key roles in the natural history of this disease. The clinical-molecular correlation depicted in this report points to the existence of important prognostic factors which extend beyond the classically utilized epithelioid versus sarcomatoid histology. Peritoneal mesothelioma remains a rare and challenging entity to treat, however, efforts such as personalized oncogenomics will continue to offer novel insights into the biology of this and other rare diseases.

## Methods

Research ethics and review board approval for Personalized OncoGenomics was granted by the University of British Columbia Research Ethics Committee. Informed written consent for molecular workup and ensuing treatment recommendations was obtained from each patient prior to commencement. Consent for surgical procedures and biopsies were obtained in the traditional manner at the institutions where the procedures were performed.

Fresh frozen biopsies were obtained by image-guided core needle biopsy (patient 1) or intraoperatively, prior to cytoreductive surgery and hyperthermic intraperitoneal chemotherapy (patient 2). Tissue was embedded in optimal cutting temperature (OCT) and serially sectioned. Intermittent hematoxylin and eosin (H&E) stained slides were reviewed for tumor content and cellularity. DNA and RNA extractions were performed to create genomic and transcriptomic libraries. DNA was similarly extracted from formalin-fixed paraffin embedded (FFPE) blocks, where available, with additional genomic library construction. Lastly, genomic libraries are created from peripherally drawn blood as reference.

Paired-end reads from all libraries were generated on an Illumina HiSeq2000 sequencer. Focused deep amplicon sequencing of both tumor specimens and blood was performed using the AmpliSeq Cancer panel (v2.0) on the Ion Torrent PGM platform.[31] A combination of established analytical pipeline and custom bioinformatic tools was used for identifying somatic nucleotide and copy number aberrations. These are described in detail in supplemental methods (S1 Text).

All imaging, procedures, and routine histology were performed in a clinical setting in accord with the standards of practice at the participating institutions. Additional ancillary tests performed outside of routine standard of practice include BAP1, MET and p16 IHC and FISH for 9p21 deletion and *MET* amplification.

Immunohistochemistry for the p16 protein was performed using CINtec E6H4 mouse monoclonal antibody (Roche; Basel, Switzerland). Primary antibody was diluted 1:3 in addition to factory dilution. Heat induced epitope retrieval was performed in CC1 buffer for 32 minutes. Visualization was with Ventana Optiview DAB (Roche, Basel, Switzerland) on the Ventana Benchmark system.

Immunohistochemical staining for MET was performed using the rabbit monoclonal antibody SP44 (Ventana medical systems; Tuscon, USA). No additional dilution was used, and heat-induced epitope retrieval was performed in CC1 buffer. Visualization was carried out using the Ventana Ultramap DAB Ventana Discovery XT system.

IHC for BAP1 was performed as previously described [32] using mouse monoclonal anti-human BAP1 clone C-4 (Santa Cruz Biotechnology, Inc., Dallas, TX), which was used at 1:400 on slides pretreated for 20 minutes in a steamer in pH 9 Tris-EDTA buffer. BAP1 was detected using the Ultravision detection system (Thermo Scientific, Waltham, MA). BAP1 staining produces a nuclear signal in all non-neoplastic cells, creating a useful internal control. BAP1 loss was identified by a homogenous loss of nuclear staining in tumor cells. Non-nuclear staining was disregarded. All slides were scored as either BAP1 lost or intact.

FISH for *CDKN2A* (p16) and MET were performed on 4 micron sections from FFPE blocks and visualized using a Metasystems Fluorescence microscope platform as previously described[33], using a *CDKN2A* (p16) gene probe cocktail and a *MET* gene probe cocktail (Cymogen DX, New Windsor, NY). The *CDKN2A* gene probe cocktail is consists of an orange fluor labeled ~298kB probe that spans the *CDKN2A* gene at 9p21.3 and a green fluor labeled centromeric chromosome 9 (CC9) probe. The *MET* gene probe cocktail consists of an orange fluor labeled ~440 kB probe that spans the *MET* gene at 7q31.2 and a green fluor labeled centromeric chromosome 7 (CC7) probe. For *CDKN2A*, 50–100 cells are scored for a homozygous deletion pattern (0 orange and 1–2 green signals) and the threshold for homozygous deletion is 12% positive cells. For *MET*, the orange and green signals for 60 cells are counted and the MET:CC7 ratio is calculated. The threshold for *MET* amplification is a MET:CC7 ratio≥2.0. For a more detailed description, see S1 Text.

## Supporting Information

S1 Fig Affected Cell-Signaling Pathways—Patient 1. Pathway diagram for patient 1, diagram based upon mutations, copy number alterations, and RNA expression data.
(TIFF)

S2 Fig Affected Cell-Signalling Pathways—Patient 2. Pathway diagram for patient 2, diagram based upon mutations, copy number alterations, and RNA expression data.
(TIFF)

S1 File Patient 1 Molecular Data. Complete list of molecular and cytogenetic alterations and RNA expression profile for patient 1 (fresh frozen biopsy of omental lesion).
(XLSX)

S2 File Patient 2 Molecular Data. Complete list of all molecular and cytogenetic alterations and RNA expression profile for patient 2 (vaginal tumor data derived from FFPE archival material, peri-colic tumor data derived from fresh-frozen biopsy).
(XLSX)

S1 Text Supplemental Methods. Detailed materials and methods utilized for personalized oncogenomic workup.
(DOC)
