# Supplementary figures and images for: Personalized Oncogenomics: Clinical Experience with Malignant Peritoneal Mesothelioma Using Whole Genome Sequencing
Source: PLoS One. 2015 Mar 23;10(3):e0119689. doi: 10.1371/journal.pone.0119689 (PMC4370594; doi:10.1371/journal.pone.0119689)

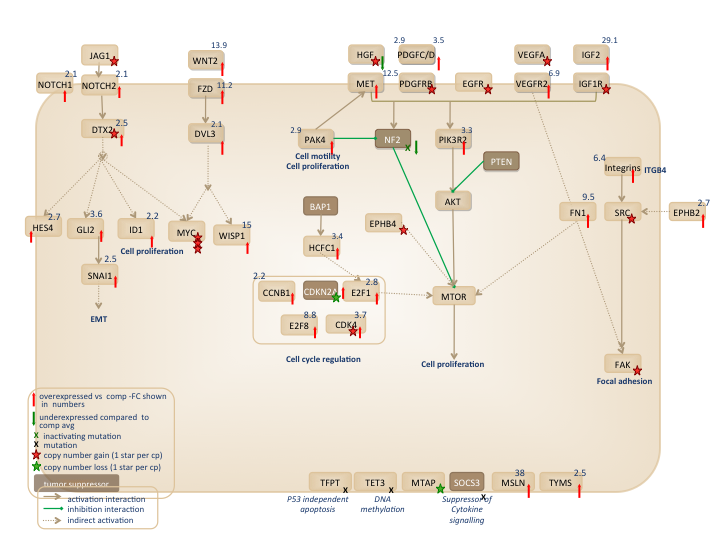

Supplement: S1 Fig — Pathway diagram for patient 1, diagram based upon mutations, copy number alterations, and RNA expression data. (TIFF) [file pone.0119689.s001.tiff]

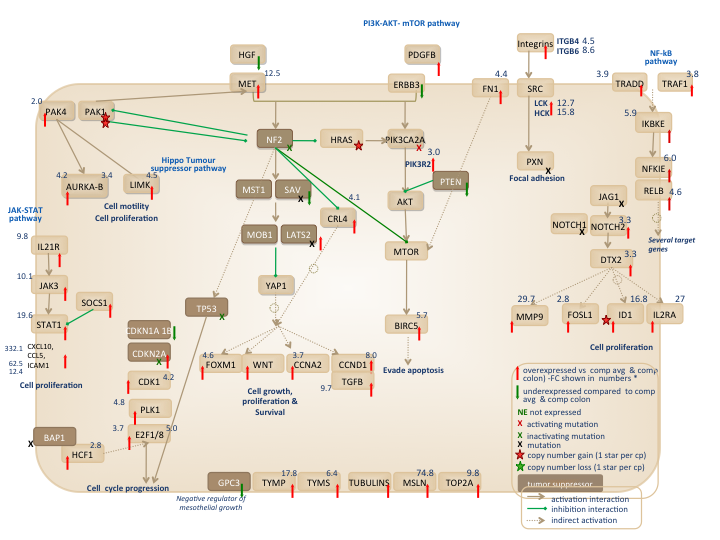

Supplement: S2 Fig — Pathway diagram for patient 2, diagram based upon mutations, copy number alterations, and RNA expression data. (TIFF) [file pone.0119689.s002.tiff]
