# Supplementary material for: Personalized Oncogenomics: Clinical Experience with Malignant Peritoneal Mesothelioma Using Whole Genome Sequencing
Source: PLoS One. 2015 Mar 23;10(3):e0119689. doi: 10.1371/journal.pone.0119689 (PMC4370594; doi:10.1371/journal.pone.0119689)
Supplement: S1 Text — Detailed materials and methods utilized for personalized oncogenomic workup. (DOC) [file pone.0119689.s005.doc]

**Supplementary Materials and Methods**

Biopsy specimens were collected from the patients in the usual clinical manner. Periferal blood was drawn as a germline DNA reference. In the case of fresh frozen biopsies performed for the purpose of molecular workup, specimens were embedded in optimal cutting temperature (OCT) compound and sectioned for RNA and DNA extraction. Intermittent sections were stained with haematoxylin and eosin and evaluated by a pathologist for tumor content and cellularity. Using 1ug DNA from the tumor and blood, we constructed two genome libraries using the Illumina TruSeq PCR free protocol (FC-121-3001). In brief, the protocol followed the same steps as Illumina genomic library construction except the size selection was done with Ampure XP beads, and the final PCR step was eliminated. Paired-end 100bp reads were generated on an Illumina HiSeq2500 sequencer following manufactures protocol with minor variations. Software version utilized was HCS1.5.8.

One transcriptome library was constructed using 1.5-3.5ug RNA from the tumor-derrived biopsy by following the strand specific RNA-Seq protocol [[[1]](#endnote-2)], with a few modifications. Briefly, PolyA+ RNA was purified using the MultiMACS mRNA isolation kit on the MultiMACS 96 separator (Miltenyi Biotec, Germany). The eluted PolyA+ RNA was ethanol precipitated and re-suspended in 10µL of DEPC treated water. First-strand cDNA was synthesized from the purified polyA+ RNA using the Superscript cDNA Synthesis kit (Life Technologies, USA) and random hexamer primers at a concentration of 5µM along with a final concentration of 1ug/ul Actinomycin D. The second strand cDNA was synthesized following the Superscript cDNA Synthesis protocol by replacing the dTTP with dUTP in dNTP mix, allowing the second strand to be digested by UNG (Uracil-N-Glycosylase, Life Technologies, USA) post adapter ligation to achieve strand specificity. Library construction was carried out by following Illumina paired end library protocol, the adapter-ligated products were purified using Ampure XP SPRI beads (Beckman-Coulter, USA), and digested with UNG (1U/ul) at 37°C for 30 min followed by deactivation at 95°C for 15 min. The digested cDNA was purified using Ampure XP SPRI beads, and then PCR-amplified with Phusion DNA Polymerase (Thermo Fisher Scientific Inc. USA) using Illumina’s PE primer set, with cycle condition 98˚C 30sec followed by 10 cycles of 98˚C 10 sec, 65˚C 30 sec and 72˚C 30 sec, and then 72˚C 5min. Paired-end 75bp reads were generated on Illumina HiSeq2500 following manufactures protocol with minor variations. Software version utilized was HCS1.5.8.

Reads were aligned to the human reference genome (GRCh37, available from[[[2]](#endnote-3)]) using BWA (0.5.7)[[[3]](#endnote-4)]. Reads from multiple lanes were merged and duplicate marked using Picard (v1.38)[[[4]](#endnote-5)]. Variants were called using mpileup (SAMtools v0.1.17)[[[5]](#endnote-6)] and a subsequently filtered with varFilter. Each tumor sample was compared to the normal sample to identify somatic copy number variants using a Hidden-Markov model based approach (CNAseq v0.0.6, described in Method of [[[6]](#endnote-7)]), LOH events (APOLLOH v0.1.1)[[[7]](#endnote-8)], single nucleotide variants using a probabilistic joint calling approach (SAMtools v0.1.17 [Error: Reference source not found], MutationSeq v1.0.2 [[[8]](#endnote-9)], Strelka v0.4.6.2 [[[9]](#endnote-10)]), and small insertions and deletions (Strelka v0.4.6.2 [Error: Reference source not found]). Variants were annotated to genes using the Ensembl database (v69) [[[10]](#endnote-11)].

RNA-Seq reads were analysed with Jaguar [[[11]](#endnote-12)] to include alignments to a database of exon junction sequences and subsequent repositioning onto the genomic reference. RNA-Seq data was processed using in-house coverage analysis software using the ‘stranded’ option to determine gene and exon read counts and normalized expression level to quantitate the level of expression for each gene and exon as normalized reads per kilobase per million observations (RPKM)[[[12]](#endnote-13)]. Expressed variants were called with SNVMix2 (v0.12.1-rc1)[[[13]](#endnote-14), [[14]](#endnote-15)] and SAMtools (v0.1.13). In the absence of RNA from matched normal tissue, we took a similar approach to Jones *et al.*[Error: Reference source not found] in conducting the differential expression analysis. Briefly, we compared the expression of genes from the tumor transcriptome library to a compendium of 19 normal transcriptomes taken from the Illumina Body Map 2.0 project (available from ArrayExpress, query ID: E-MTAB-513)[[[15]](#endnote-16)]. This compendium is comprised of 16 different tissue types. This approach allows for discovering tumor-specific changes in expression and thus provides a better understanding of the mechanism of the disease as well as opportunities in identifying relevant therapeutic interventions. Number of reads per kilobase of exon model per million mapped reads (RPKM value)[Error: Reference source not found] calculated for each protein coding gene as annotated in Ensembl (v59)[[[16]](#endnote-17)] was used as a measure of expression. Differential expression analysis was done using outlier statistics and fold change comparison between the tumor sample RPKM and the compendium’s mean RPKM for each gene. Overexpressed genes were defined as having a Benjamini and Hochberg [[[17]](#endnote-18)] corrected outlier *P*-value < 0.05 and fold change > 2. Genes with an uncorrected outlier *P*-value < 0.1 and fold change < -2 were considered underexpressed. Both genomic and RNA-Seq tumour data were also assembled using Trans-ABySS (v1.4.3)[Error: Reference source not found] to identify structural variants and fusion genes.

Nonsynonymous coding SNVs were annotated using the COSMIC database[[[18]](#endnote-19)] to search for previously observed somatic mutations. Genes were linked to cancer pathways using KEGG [[[19]](#endnote-20)] and Ingenuity Pathway Analysis [[[20]](#endnote-21)], known cancer genes using the COSMIC cancer gene census[Error: Reference source not found] and Uniprot knowledgebase[[[21]](#endnote-22)], and drugs using DrugBank[[[22]](#endnote-23)]and the Therapeutic Target Database[[[23]](#endnote-24)].

**Supplemental References**

1. [?] Parkhomchuk D, Borodina T, Amstislavskiy V, Banary M, Hallen L, Krobitsch S, Lehrach H, Soldatov A. (2009) Transctiptome analysis by strand-specific sequencing og complementary DNA. Nucleic Acids Res. 37(18):e123. [↑](#endnote-ref-2)
2. [?] (2011) Human Reference Genome Available online at: http://wwwbcgscca/downloads/genomes/9606/hg19/1000genomes/bwa_ind/genome [Accessed Sept 1 2014]. [↑](#endnote-ref-3)
3. [?] H Li and R Durbin (2010) Fast and Accurate Long-Read Alignment with Burrows-Wheeler Transform Bioinformatics 26(5): 589-95. [↑](#endnote-ref-4)
4. [?] (2014) *Picard* Http://Picard.Sourceforge.Net/ [Accessed Sept 1 2014]. [↑](#endnote-ref-5)
5. [?] H Li, B Handsaker, A Wysoker, T Fennell, J Ruan, N Homer, G Marth, G Abecasis, and R Durbin (2009) The Sequence Alignment/Map Format and Samtools, Bioinformatics 25(16): 2078-9. [↑](#endnote-ref-6)
6. [?] SJ Jones, J Laskin, YY Li, OL Griffith, J An, M Bilenky, YS Butterfield, T Cezard, E Chuah, R Corbett, AP Fejes, M Griffith, J Yee, M Martin, M Mayo, Melnyk, RD Morin, TJ Pugh, T Severson, SP Shah, M Sutcliffe, A Tam, J Terry, N Thiessen, T Thomson, R Varhol, T Zeng, Y Zhao, RA Moore, DG Huntsman, I Birol, M Hirst, RA Holt, and MA Marra. (2010) Evolution of an Adenocarcinoma in Response to Selection by Targeted Kinase Inhibitors. Genome Biol 11(8): R82. [↑](#endnote-ref-7)
7. [?] G Ha, A Roth, D Lai, A Bashashati, J Ding, R Goya, R Giuliany, J Rosner, A Oloumi, K Shumansky, SF Chin, G Turashvili, M Hirst, C Caldas, MA Marra, S Aparicio, and SP Shah. (2012) Integrative Analysis of Genome-Wide Loss of Heterozygosity and Monoallelic Expression at Nucleotide Resolution Reveals Disrupted Pathways in

   Triple-Negative Breast Cancer. Genome Res 22(10): 1995-2007. [↑](#endnote-ref-8)
8. [?] J Ding, A Bashashati, A Roth, A Oloumi, K Tse, T Zeng, G Haffari, M Hirst, MA Marra, A Condon, S Aparicio, SP Shah. (2012) Feature-Based Classifiers for Somatic Mutation Detection in Tumour-Normal Paired Sequencing Data. Bioinformatics 28(2): 167-75. [↑](#endnote-ref-9)
9. [?] CT Saunders, WS Wong, S Swamy, J Becq, LJ Murray, RK Cheetham. (2012) Strelka: Accurate Somatic Small-Variant Calling from Sequenced Tumor-Normal Sample Pairs. Bioinformatics 28(14): 1811-7. [↑](#endnote-ref-10)
10. [?] P Flicek, I Ahmed, MR Amode, D Barrell, K Beal, S Brent, D Carvalho-Silva, P Clapham, G Coates, S Fairley, S Fitzgerald, L Gil, C Garcia-Giron, L Gordon, T Hourlier, S Hunt, T Juettemann, AK Kahari, S Keenan, M Komorowska, E Kulesha, I Longden, T Maurel, WM McLaren, M Muffato, R Nag, B Overduin, M Pignatelli, B Pritchard, E Pritchard, HS Riat, GR Ritchie, M Ruffier, M Schuster, D Sheppard, D Sobral, K Taylor, A Thormann, S Trevanion, S White, SP Wilder, BL Aken, E Birney, F Cunningham, I Dunham, J Harrow, J Herrero, TJ Hubbard, N Johnson, R Kinsella, A Parker, G Spudich, A Yates, A Zadissa, SM Searle (2013) Ensembl 2013. Nucleic Acids Res 41(1): D48-55. [↑](#endnote-ref-11)
11. [?] Y Butterfield, R Corbett, N Thiessen, A He, I Birol, SJM Jones, MA Marra. (2012) JAGuaR: Junction Alignments to Genome for Repositioning of RNA-seq Reads Available online at: http://wwwbcgscca/platform/bioinfo/docs/jaguar/Butterfield_JAGuaR_Nov2011pdf/view [Accessed Sept 1 2014]. [↑](#endnote-ref-12)
12. [?] A Mortazavi, BA Williams, K McCue, L Schaeffer, B Wold. (2008)Mapping and Quantifying Mammalian Transcriptomes by Rna-Seq. Nat Methods*,* 5(7): 621-8. [↑](#endnote-ref-13)
13. [?] R Goya, MG Sun, RD Morin, G Leung, G Ha, KC Wiegand, J Senz, A Crisan, MA Marra, M Hirst, D Huntsman, KP Murphy, S Aparicio, SP Shah. (2010) Snvmix: Predicting Single Nucleotide Variants from Next-Generation Sequencing of Tumors. Bioinformatics*,* 26(6): 730-6. [↑](#endnote-ref-14)
14. [?] SP Shah, RD Morin, J Khattra, L Prentice, T Pugh, A Burleigh, A Delaney, K Gelmon, R Guliany, J Senz, C Steidl, RA Holt, S Jones, M Sun, G Leung, R Moore, T Severson, GA Taylor, AE Teschendorff, K Tse, G Turashvili, R Varhol, RL Warren, P Watson, Y Zhao, C Caldas, D Huntsman, M Hirst, MA Marra, S Aparicio. (2009) Mutational Evolution in a Lobular Breast Tumour Profiled at Single Nucleotide Resolution. *Nature,* 461(7265): 809-13 [↑](#endnote-ref-15)
15. [?] YW Asmann, BM Necela, KR Kalari, A Hossain, TR Baker, JM Carr, CDavis, JE Getz, G Hostetter, X Li, SA McLaughlin, DC Radisky, GP Schroth, HE Cunliffe, EA Perez, EA Thompson. (2012) Detection of Redundant Fusion Transcripts as Biomarkers or Disease-Specific Therapeutic Targets in Breast Cancer. Cancer Res 72(8): 1921-8.

    [↑](#endnote-ref-16)
16. [?] TJ Hubbard, BL Aken, S Ayling, B Ballester, K Beal, E Bragin, S Brent, Y Chen, P Clapham, L Clarke, G Coates, S Fairley, S Fitzgerald, J Fernandez-Banet, L Gordon, S Graf, S Haider, M Hammond, R Holland, K Howe, A Jenkinson, N Johnson, A Kahari, D Keefe, S Keenan, R Kinsella, F Kokocinski, E Kulesha, D Lawson, I Longden, K Megy, P Meidl, B Overduin, A Parker, B Pritchard, D Rios, M Schuster, G Slater, D Smedley, W Spooner, G Spudich, S Trevanion, A Vilella, J Vogel, S White, S Wilder, A Zadissa, E Birney, F Cunningham, V Curwen, R Durbin, XM Fernandez-Suarez, J Herrero, A Kasprzyk, G Proctor, J Smith, S Searle, P Flicek. (2009) Ensembl 2009. Nucleic Acids Res 37 (1): D690-7. [↑](#endnote-ref-17)
17. [?] Y Klipper-Aurbach, M Wasserman, N Braunspiegel-Weintrob, D Borstein, S Peleg, S Assa, M Karp, Y Benjamini, Y Hochberg, Z Laron. (1995) Mathematical Formulae for the Prediction of the Residual Beta Cell Function During the First Two Years of Disease in Children and Adolescents with Insulin-Dependent Diabetes Mellitus. Med Hypotheses 45 (5): 486-90. [↑](#endnote-ref-18)
18. [?] SA Forbes, N Bindal, S Bamford, C Cole, C Y Kok, D Beare, M Jia, R Shepherd, K Leung, A Menzies, JW Teague, PJ Campbell, MR Stratton, PA Futreal. (2011) Cosmic: Mining Complete Cancer Genomes in the Catalogue of Somatic Mutations in Cancer. Nucleic Acids Res 39 (1): D945-50. [↑](#endnote-ref-19)
19. [?] M Kanehisa, S Goto, Y Sato, M Furumichi, M Tanabe. (2012) Kegg for Integration and Interpretation of Large-Scale Molecular Data Sets. Nucleic Acids Res 40 (1): D109-14. [↑](#endnote-ref-20)
20. [?] The pathways and networks were generated through the use of QIAGEN’s Ingenuity Pathway Analysis (IPA®,QIAGEN Redwood City, wwwqiagencom/ingenuity) [accessed Sept 1 2014]. [↑](#endnote-ref-21)
21. [?] M Magrane and U Consortium. (2011) Uniprot Knowledgebase: A Hub of Integrated Protein Data. Database (Oxford) bar009. [↑](#endnote-ref-22)
22. [?] DS Wishart, C Knox, AC Guo, D Cheng, S Shrivastava, D Tzur, B Gautam, M Hassanali. (2008) Drugbank: A Knowledgebase for Drugs, Drug Actions and Drug Targets. *Nucleic Acids Res,* 36 (1): D901-6 . [↑](#endnote-ref-23)
23. [?] F Zhu, Z Shi, C Qin, L Tao, X Liu, F Xu, L Zhang, Y Song, J Zhang, B Han, P Zhang Y Chen. (2012) Therapeutic Target Database Update 2012: A Resource for Facilitating Target-Oriented Drug Discovery. Nucleic Acids Res. 40 (1): D1128-36. [↑](#endnote-ref-24)
